# The heterogeneity of regional cortical atrophy in early Alzheimer’s disease

**DOI:** 10.1002/alz.092522

**Published:** 2025-01-03

**Authors:** Frederick A Page, Shayam Suseelan, Gayatri Kulkarni, Joseph Nowell, Paul Edison

**Affiliations:** ^1^ Imperial College London, London United Kingdom; ^2^ Imperial College London University, London United Kingdom; ^3^ Cardiff University, Wales United Kingdom; ^4^ Division of Neurology, Department of Brain Sciences, Imperial College London, United Kingdom, London, London United Kingdom

## Abstract

**Background:**

Cortical atrophy in Alzheimer’s disease (AD) is typically thought to commence in the medial temporal lobe. However, growing evidence suggests that this may not be true for all patients. Here, we explored atrophy patterns in patients with mild cognitive impairment (MCI) and AD and whether atrophy patterns are associated with specific clinical and neuropathological outcomes.

**Methods:**

1124 AD and MCI patients were selected from the Alzheimer’s Disease Neuroimaging Initiative. Baseline 3T magnetic resonance imaging scans were processed for all patients who were then subtyped based on regions of predominant atrophy. Baseline cognitive performance and rates of cognitive decline over one year were compared between atrophy subtypes. Regional tau pathology was also evaluated with [18F] AV1451 positron emission tomography.

**Results:**

The following atrophy subtypes were identified: medial‐temporal dominant, frontal‐dominant, parietal‐dominant, diffuse, and occipital‐dominant. Baseline cognitive performance and tau were indicated to differ between subtypes.

**Conclusion:**

Initial cortical atrophy in AD and MCI may be highly heterogeneous. Additionally, since atrophy subtypes showed unique cognitive performances and tau distributions, they may respond differently to treatment. This study provides grounds for an increased research focus on cortical atrophy in regions outside the medial temporal lobe, especially the parietal lobe.

